# Early response of monocyte-derived macrophages from vaccinated and non-vaccinated goats against in vitro infection with *Mycobacterium avium* subsp. *paratuberculosis*

**DOI:** 10.1186/s13567-021-00940-y

**Published:** 2021-05-12

**Authors:** Noive Arteche-Villasol, Daniel Gutiérrez-Expósito, Raquel Vallejo, Jose Espinosa, Natalia Elguezabal, Iraia Ladero-Auñon, Marcos Royo, María del Carmen Ferreras, Julio Benavides, Valentín Pérez

**Affiliations:** 1 Departamento de Sanidad Animal, Facultad de Veterinaria, Universidad de León, Campus de Vegazana s/n, 24007 León, Spain; 2 Instituto de Ganadería de Montaña (CSIC-ULE),, Finca Marzanas-Grulleros, 24346 León, Spain; 3 Departamento de Sanidad Animal, NEIKER-Instituto Vasco de Investigación y Desarrollo Agrario, Berreaga 1, Derio, 48169 Bizkaia, Spain

**Keywords:** *Map*, Vaccination, Caprine monocyte-derived macrophages, Viability, Phagocytosis

## Abstract

Paratuberculosis is a disease of ruminants caused by *Mycobacterium avium* subsp. *paratuberculosis* (*Map*). Vaccination is the most cost-effective control method. However, despite the fact that macrophages are the main target cells for this pathogen, the precise mechanisms behind the response of the macrophage to *Map* infection and how it is modified by vaccination are yet poorly understood. The aim of this study was to investigate the effect of Silirum^®^ vaccination in the early immune response of caprine monocyte-derived macrophages (CaMØs). Peripheral blood mononuclear cells (PBMCs) were obtained from vaccinated and non-vaccinated goats, cultured in vitro until differentiation to macrophages and infected with *Map*. After a 24 h incubation, *Map* viability and DNA were assessed in culture by viable colony count and real time quantitative polymerase chain reaction (qPCR). In addition, *Map* phagocytosis and expression of IL-10, IL-12, IFN-γ, TNF-α, IL-17A, IL-1β, iNOS, IL-6 and MIP-1β were also evaluated through immunofluorescence labelling and reverse transcriptase qPCR (RT-qPCR), respectively. A significant reduction of *Map* viability was observed in both supernatants (*P* < 0.05) and CaMØs (*P* < 0.001) from the vaccinated group. Similarly, the percentage of infected CaMØs and the number of internalized *Map* by CaMØs (*P* < 0.0001) was higher in the vaccinated group. Finally, iNOS (*P* < 0.01) and IL-10 were significantly up-regulated in CaMØs from vaccinated goats, whereas only MIP-1β was up-regulated in non-vaccinated animals (*P* < 0.05). These results show that vaccination modifies the immune response of CaMØs, suggesting that the phagocytosis and microbiocidal activity of macrophages against *Map* is enhanced after vaccination.

**Supplementary Information:**

The online version contains supplementary material available at 10.1186/s13567-021-00940-y.

## Introduction

Paratuberculosis, a disease characterized by the development of a chronic granulomatous enteritis, is caused by the intracellular pathogen *Mycobacterium avium* subspecies *paratuberculosis* (*Map*) that affects domestic ruminants such as cattle, sheep and goats and may cause weight loss, reduced milk production and premature culling [1, 2]. Animals are usually infected when they are young because of a high degree of environmental contamination [3, 4] but clinical signs, are usually not developed until adulthood. Nonetheless, most of the infected animals remain subclinical for their entire life [5].

Vaccination against paratuberculosis is among the most efficient measures for reducing the incidence of clinical cases [6]. To date, different types of vaccines have been evaluated, from killed whole-cell-based vaccines to attenuated vaccines or those more recent, made from recombinant protein or DNA [7]. The only heat-killed mycobacteria vaccines commercially available (Gudair® and Silirum^®^) are not able to confer sterile immunity but they have shown important benefits in reducing the productive losses and environmental contamination [6, 8].

Vaccines are focused on a rapid and effective stimulation of a cell-mediated immune response [8, 9] although the specific mechanisms that explain how this modulation occurs are yet unknown [10]. The ability to control the disease has been associated with the development of a Th1-type immune response mediated by CD4^+^ lymphocytes and the classical activation (M1) of macrophages [11, 12]. In this context, macrophages are the target cells where *Map* is able to survive and multiply [13] and play a crucial role in the host–pathogen interaction as they are responsible for *Map* elimination [14]. Specifically, after penetrating the intestinal epithelial barrier, *Map* is phagocytosed by sub-epithelial macrophages, stimulating the recruitment of lymphocytes and monocytes in situ and the development of focal granulomas [15]. Initial stages of *Map* infection are characterized by a strong cell-mediated immune response via stimulation of IFN-γ by CD4^+^ lymphocytes that induces classical activation of macrophages (M1), releasing pro-inflammatory cytokines (e.g. TNF-α, IL-1β and IL-6) and increasing microbicidal activity [16–18]. However, progression of the disease shifts to a Th2-type immune response where macrophages are predominantly alternatively activated (M2) by the stimulation of IL-10 and IL-4 [19, 20], leading to the up-regulation of anti-inflammatory cytokines such as TGF-β or IL-10 and favouring the intracellular survival and growth of *Map* [17, 18].

Due to the importance of macrophages in the immune response against paratuberculosis, peripheral blood monocyte-derived macrophages (MDMs) have emerged as a relevant in vitro experimental model to study the immune response (phagocytosis, growth and cytokine production) against *Map* infection [13, 21, 22]. Survival of *Map* inside macrophages lies on the ability to modify the intracellular environment so as to prevent its destruction [23], for example, through the interference of phagolysosome maturation or acidification [24]. Besides that, *Map* may be involved in the modification of gene expression profiles that could limit microbicidal response [25].

Despite their key role in the immune response against *Map*, and the proved influence of vaccination on this response, studies addressing the modulation of macrophages by paratuberculosis vaccination are scarce [26], especially those conducted using MDMs models. For this reason, the aim of this study was to analyse whether vaccination could modify the phagocytic activity and immune response of caprine MDMs against *Map*.

## Materials and methods

### Ethics statement

All the procedures were approved by the Ethics Committee of the Instituto de Ganadería de Montaña (IGM, CSIC-ULE) and the Subcommittee on Animal Experiments and Welfare of the University of León (ULE). Handing and sampling procedures were designed according to European (86/609) and Spanish laws (R.D. 223/1988, R.D. 1021/2005, R.D. 53/2013) and were minimized in order to reduce stress and the health risks of the animals and personnel involved.

### Animals

Twelve one-month-old goat female kids (murciano-granadina breed) were selected from a flock without clinical cases, tested negative to paratuberculosis in the last five years and housed in the facilities of the Instituto de Ganadería de Montaña (IGM, CSIC-ULE) in Grulleros, León. After one month of adaptation period, animals were tested against *Map* infection by indirect enzyme-linked immunoabsorbent assay (ID Screen^®^ Paratuberculosis indirect, IDVet, Grabels, France) and IFN-γ release test (Bovigam^®^ *Mycobacterium bovis* IFN-γ test for cattle, Thermo Fisher Scientific, Waltham, USA). Both commercial kits have been previously used and standardized for diagnosis of ovine and caprine paratuberculosis [27–29]. Once established that animals did not have antibodies against *Map* and were negative to the IFN-γ release test, they were separated into two groups: non-vaccinated (*n* = 6) and vaccinated (*n* = 6). At the age of two months, vaccination was performed by a subcutaneous injection in the brisket with 1 mL of commercial vaccine Silirum^®^ which contained 2.5 mg of heat-killed 316F *Map* strain plus Montanide mineral oil as adjuvant (CZVaccines, Porriño, Spain). Heparinized-blood samples of each animal were collected 30 days after vaccination to generate caprine monocyte-derived macrophages (CaMØs) in order to perform the in vitro assays. This response window was selected based on our previous observations in vaccinated goat kids that showed an increase in the IFN-γ response from that time onwards (data not shown).

### In vitro generation of caprine monocyte-derived macrophages

CaMØs were generated as previously described [30]. Briefly, 200 mL of peripheral blood of each animal was collected from the jugular vein into lithium heparin Vacutainer™ tubes (Becton Dickinson and Company, UK). Peripheral blood mononuclear cells (PBMCs) were separated by gradient density centrifugation on Lymphoprep™ (STEMCELL Technologies^®^, Cologne, Germany), seeded in cell culture flasks (Labbox, Barcelona, Spain) at a density of 10^7^ cells/mL in 10 mL of supplemented RPMI1640 medium + GlutaMax™ (Gibco, Paisley, UK), and incubated for 3 h at 37 °C and 5% CO_2_ in a humidified incubator. Later on, non-adherent cells were removed and 10 mL of fresh supplemented RPMI16040 medium with 60 ng/mL of caprine GM-CSF (KingFisher Biotech^®^, MN, USA) was added. At the third day of culture, flasks were washed twice with warm PBS 1× and medium was replaced by fresh supplemented RPMI medium. Adherent monocytes were allowed to differentiate for the next 4 days to CaMØs which were checked under an inverted microscope (LEITZ DM IL, Leica, Wetzlar, Germany). At day 7 of culture and once CaMØs complied with the morphological and phenotypic characteristics described by Arteche-Villasol et al. [30], cells were harvested using ice-cold PBS 1× with 2 mM EDTA and soft scraping and reseeded at a density of 10^5^ cells/mL in supplemented RPMI medium with GM-CSF in 24-well culture plates and 24-well culture plates with sterile glass coverslips of 13 mm diameters (VWR, Darmstadt, Germany). The purity of CaMØs (> 90%) was determined by flow cytometry using CD14, MHC-II and CD11b antibodies [30]. Then, CaMØs were allowed to adhere for 24 h in the same conditions mentioned above.

### Bacteria culture and CaMØs infection

*Map* strain K10, a standardized reference bovine type strain, was provided by NEIKER (Basque Institute for Agricultural Research and Development, Derio, Spain). The organisms were grown in 7H9 broth supplemented with 10% oleic acid-albumin-dextrose-catalase enrichment (OADC) (Becton Dickinson and Company, MD, USA), 0.2% glycerol, 0.05% Tween 80 (Panreac Quimica SA, Barcelona, Spain) and 2 mg/L of mycobactin J (Allied Monitor, Fayette, MO) (7H9-OADC-MJ) to exponential phase for 3 weeks at 37 ± 1 °C. Bacterial suspension was adjusted at a concentration of 10^8^ *Map* CFU/mL in glycerol: water (1:1) after colony forming units (CFU) estimation by optical density and colony count in agar-solidified 7H9 with OADC, glycerol and mycobactin J in quadruplicate to assess the CFU per mL in the inoculum. Afterwards, bacterial suspension was frozen at −80 °C until use in the next three weeks. Prior to CaMØs infection, aliquots were thawed in fresh 7H9-OADC-MJ medium and incubated for 3 h at 37 °C. Then, bacterial suspensions were centrifuged at 3000 × *g* for 10 min and bacterial pellets were washed twice with PBS 1× and resuspended in PBS 1× and passed through a 30-gauge needle and vigorously vortexed to disperse clumps before infection [31].

Prior to infection, CaMØs, including control wells, were washed twice with warm PBS 1× and freshly supplemented RPMI1640 medium without antibiotics were added to wells. Then, CaMØs were infected with *Map* at a multiplicity of infection (MOI) 10:1 (10 bacilli/macrophage) [32]. Thereupon, plates were incubated at 37 °C and 5% CO_2_ in a humidified incubator for 24 h as previously described [32]. For each animal, supernatants and CaMØs were tested for (i) *Map* viability by CFU count and (ii) DNA quantification by qPCR in independent wells using duplicates for each test. In addition, a third and a fourth sample of CaMØs were assessed for (iii) cytokines and iNOS expression by RT-qPCR and (iv) quantification of intracellular *Map* by epifluorescence microscopy by duplicate (Additional file 1).

For *Map* viability, supernatants were collected and centrifuged at 10 000 × *g* for 10 min, washed twice with PBS 1×, resuspended in 200 µL of PBS 1× and stored at 4 °C until use. Then CaMØs were scraped and collected in 500 µL of PBS 1× and stored at −20 °C until use.

For *Map*-DNA quantification by real time qPCR, supernatants were collected and stored at −20 °C whereas CaMØs were processed as mentioned above for viability assay.

CaMØs seeded onto sterile glass coverslips of 13 mm diameters (VWR, Darmstadt, Germany) for *Map* quantification by epifluorescence microscopy were fixed and permeabilized with 500 µL methanol (Fisher Chemical™, UK) for 20 min at −20 °C and washed twice with PBS 1×. Then, fixed CaMØs were stored with 2 mL of PBS 1× per well at 4 °C until staining.

Collected samples for *Map* viability, DNA quantification and epifluorescence microscopy were processed within a week.

Finally, for the study of gene expression levels, CaMØs were lysed by adding 350 µL of RLT buffer per well (RNeasy Mini Kit, Quiagen, Hilden, Germany) for subsequent RNA isolation following manufacturer indications.

### *Map* viable count

Viable count of *Map* was performed in both collected supernatants and CaMØs. Briefly, CaMØs were centrifuged and the cell pellet was lysed by vigorous vortexing for 10 s with 500 µL of 0.1% Triton X-100. Two 10-fold serial dilutions in PBS of cell lysates and their respective supernatants were grown by spreading 100 µL of the samples in triplicate on 7H9-OADC-MJ-T agar plates before incubation at 37 °C for one month. Finally, CFUs count was performed by direct observation of the plates and total number of viable CFU was estimated for each sample.

### Quantification of *Map* by real time quantitative polymerase chain reaction

Total DNA extraction of CaMØs cultures and their respective supernatants was carried out using Maxwell^®^ 16 Cell DNA Purification Kit with the Maxwell 16 Instrument (Promega, WI, USA) following manufacturer’s protocol. Afterwards, DNA was quantified using QuantiFluor™ ONEdsDNA System kit and Quantus™ Fluoremeter (Promega, WI, USA). In order to quantify *Map* DNA in culture samples and supernatants, a standard curve was generated using genomic DNA extracted from 2 × 10^8^ *Map* bacteria.

Quantification of IS*900* sequence was performed as previously described Espinosa et al. [33] using 7500 Real-Time PCR System (Applied Biosystems™, Spain). The reaction mixture contained 0.5 µL of 250 nM of forward (MP10-1, [5′-ATGCGCCACGACTTGCAGCCT-3′]) and reverse (MP11-1, 5′-GGCACGGCTCTTGTTGTAGTCG-3′]) primers [34], 10 µL of PowerUp™ SYBR™ Green Master Mix (Applied Biosystems™, CA, USA) and 9 µL of DNA template in a final volume of 20 µL and was carried out in duplicate. Following an initial activation at 95 °C for 15 min, quantification was performed via 45 cycles of a two-step assay with denaturation at 95 °C for 30 s and annealing and amplification at 68 °C for 60 s. The standard curve for this assay was performed with 10-fold diluted samples of *Map*-genomic DNA ranging from 1000 pg to 0.001 pg/reaction. Samples were considered as positive when the dissociation peak (*Tm*) was 89.1 ± 1.5 °C and threshold cycles (*Ct*) were ≤ 37 [35, 36]. The qPCR results were analysed using 7500 Software v2.0.6 (Applied Biosystems™, Spain). Thus, the quantity of *Map* DNA (pg) of each well was calculated by interpolation of their *Ct* values with the standard curve as previously described [37] and the mean quantity was calculated from both duplicates.

### Quantification of intracellular *Map* by epifluorescence microscopy

Fixed CaMØs were washed twice with PBS 1× and *Map* was stained with polyclonal antibody anti-*Map* (Dako, CA, USA) at a dilution of 1:2000 in block buffer Animal-Free Blocker® and Diluent, R.T.U (Vector Laboratories, CA, USA) and incubated overnight at 4 °C. CaMØs were washed again and a secondary antibody goat anti-rabbit IgG Alexa Fluor^®^ 488 (ab150077, Abcam, Cambridge, UK) was added at a dilution of 1:2000 in block buffer and incubated for 45 min at room temperature in the dark. After secondary antibody incubation, wells were washed twice and CellMask (ThermoFisher Scientific, OR, USA) was added in a proportion of 1:500 and incubated for 30 min in the dark. Finally, CaMØs were washed again, removed from the wells and mounted with DAPI mounting medium (Abcam, Cambridge, UK) on glass slides and stored in the dark at 4 °C. Immunofluorescence staining was previously optimized in order to avoid non-specific binding and autofluorescence through the incubation of CaMØs with primary and secondary antibodies separately. Slide observation was performed at 400× magnification on a direct microscope (Eclipse Ni-E, Nikon, NY, USA) and using appropriate epifluorescence filters for FITC, TRITC and DAPI. Images were captured using a CMOS scientific camera (Photometrics^®^ Prime BSI™, AZ, USA) and merged by NIS-Elements software (Nikon, NY, USA). Twenty different fields were randomly selected and examined in each slide using ImageJ 1.52t (NIH) and the total number of infected and non-infected CaMØs were counted in order to estimate the percentage of infected cells. Because *Map* tendency to form clumps hampered the estimation of the number of bacteria per CaMØ [38] we followed a procedure similar to that described by Gollnick et al. [39], where three categories were established based on the size of the positive signal corresponding to *Map* bacilli (s) clump: low (2–40 µm^2^: 1–4 bacilli), medium (41–100 µm^2^: 5–10 bacilli) and high (101–200 µm^2^: 10–20 bacilli).

### Determination of cytokines and iNOS mRNA expression

Total RNA isolation from CaMØs was carried out the same day of collection using RNeasy^®^ Mini Kit (Quiagen, Hilden, Germany) following manufacturer’s steps. RNA was quantified using a QuantiFluor™ RNA System kit and Quantus™ Fluoremeter (Promega, WI, USA). Then, reverse transcription of a maximum of 2.5 µg of RNA was performed by using SuperScript™ VILO™ Master Mix (Invitrogen™, Paisley, UK) according to the manufacturer’s instructions using SimpliAmp™ Thermal Cycler (Applied Biosystems™, Warrington, UK). Finally, all cDNA samples were adjusted to 10 ng/µL by dilution in nuclease-free water and stored at −80 °C and used within two weeks.

RT-qPCR reactions were performed in a 96-well plate (Applied Biosystems™, Warrington, UK) using 10 µL of PowerUp™, SYBR™ Green master mix (Applied Biosystems™, CA, USA), 10 µM of each primer and 2 µL of diluted cDNA template on a 7500 Fast Real-Time PCR System (Applied Biosystems™, CA, USA). Primer sequences used for IFN-γ, IL-10, TNF-α, IL-12 and β-actin have been described previously (Additional file 2) [40, 41]. Primers for IL-1β, IL-17A, iNOS, IL-6 and MIP-1β were designed and checked by using Primer3Plus and Oligoanalyzer Tool (IDT™) software, respectively (Additional file 2). The mRNA expression levels were normalized using β-actin as housekeeping gene. Furthermore, amplification efficiencies were analysed including a seven-point standard curve for each target gene on every plate prepared from 10-fold serial dilutions of a starting concentration of 1 ng/µL of a conventionally prepared PCR product.

Data were analysed by using relative quantification 2^−ΔΔ*Ct*^ method as previously described Livak and Schimitten, [42]. To assess the effect of *Map* infection and vaccination on cytokine expression in CaMØs, Δ*Ct* mean value of non-infected CaMØs (C-) from non-vaccinated animals were used as calibrator for calculation of ΔΔ*Ct*. cDNA samples from control and infected CaMØs were prepared in parallel and analysed on the same real-time PCR run.

### Statistical analysis

Normality of data distribution was tested using Shapiro–Wilk test analysis where only results from cytokine expression levels were normally distributed. Non-parametric Wilcoxon signed-rank tests for related samples were conducted to estimate the differences in the *Map*-DNA quantification between supernatants and CaMØs stemming from the same group (non-vaccinated and vaccinated group). Besides, differences between non-vaccinated and vaccinated groups in supernatants and CaMØs were calculated using Mann–Whitney U test for unrelated samples.

Similarly, results from cytokine expression and logarithmic transformation of viable count were used for comparisons between supernatants and CaMØs within non-vaccinated and vaccinated groups by paired *t*-test whereas differences between groups for each type of sample were assessed by unpaired *t*-test.

Finally, the percentage of *Map-*infected cells quantified by epifluorescence microscopy was calculated by the division of the number of infected cells by the total number of CaMØs per photograph. In addition, the infection rate was calculated by dividing the number of cells from each category (low, medium, high) by the total number of infected cells. Then, comparisons between non-vaccinated and vaccinated groups were made using Mann–Whitney U test. All the statistical analyses were carried out using Graphpad Prism 6.0 software (San Diego, CA, USA), where a *P* value of < 0.05 was considered statistically significant.

## Results

### Viability of *Map* in CaMØs and supernatants

**Figure 1:**
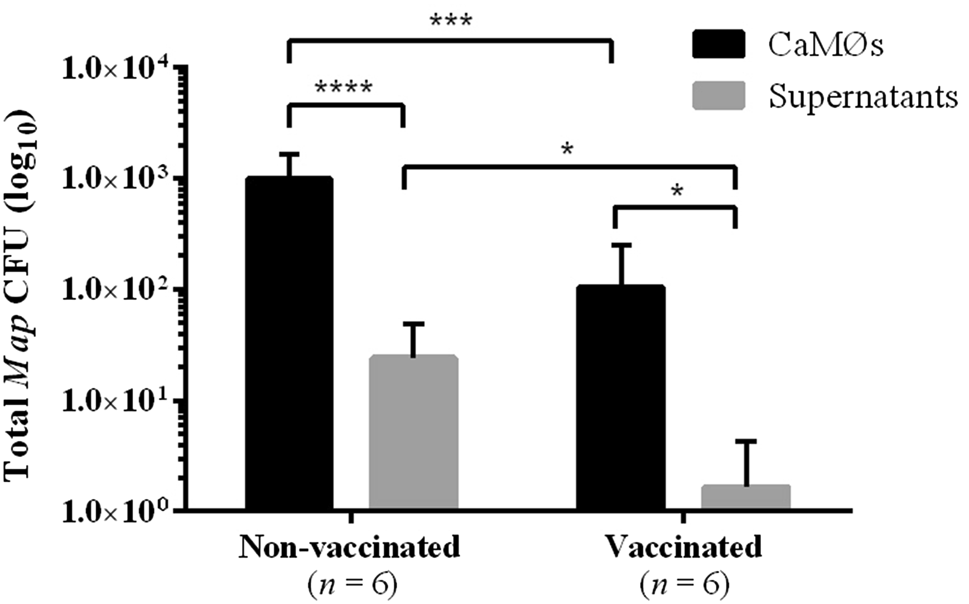
**Total viable count of internalized and free Map in CaMØs and in supernatants, respectively.** Log_10_ transformed data from non-vaccinated and vaccinated goats are represented as means values and standard deviations (*n* = 6). *p < 0.05; **p < 0.01; *** *p* < 0.001; ****p < 0.0001.

No *Map* growth was observed in any of the uninfected (C-) CaMØs from vaccinated and non-vaccinated goats. In infected CaMØs from both groups, the number of viable bacteria was higher inside CaMØs than in the supernatants. This difference was 60 and 40-fold higher in vaccinated (*P* < 0.05) and non-vaccinated (*P* < 0.0001) groups, respectively (Figure 1). When comparing the vaccinated and non-vaccinated animals, and despite large inter-individual variations, a statistically significant 10-fold reduction in the number of viable bacteria was observed in CaMØs from the vaccinated group in comparison to the non-vaccinated group (*P* < 0.001) (Figure 1). Similarly, the number of viable bacteria was also lower (15-fold reduction) in supernatants from vaccinated goats than in non-vaccinated ones (*P* < 0.05) (Figure 1) (Additional file 3).

### Quantification of *Map* DNA in CaMØs and supernatants

**Figure 2:**
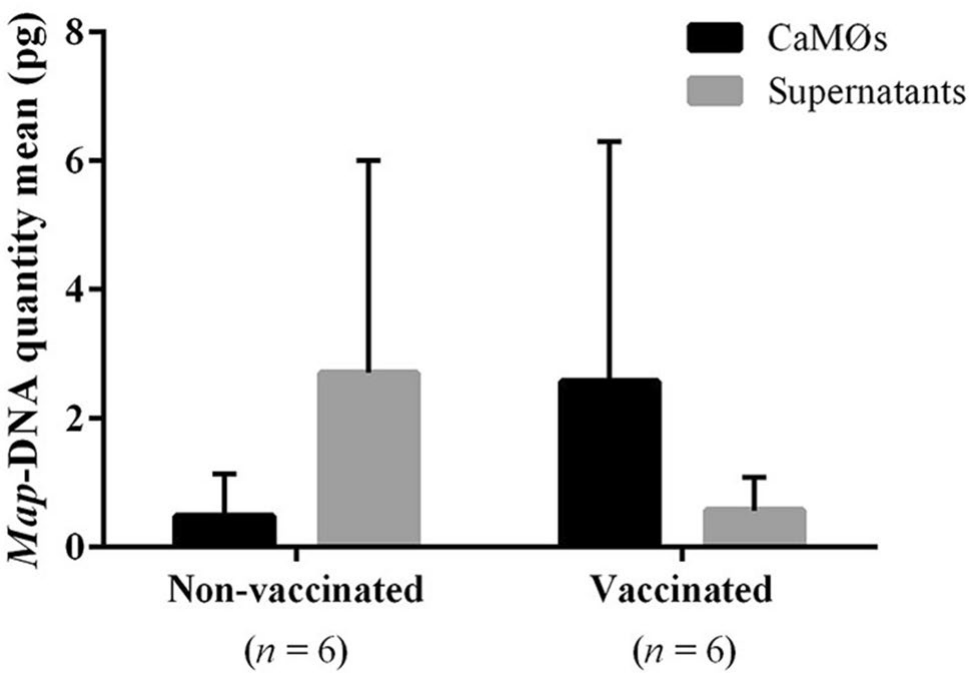
**Quantification of IS900 sequence by qPCR in CaMØs and supernatants from non-vaccinated and vaccinated goats.** Quantification was performed after 24 h of incubation with a MOI of 10:1 of *Mycobacterium avium* subsp. *Paratuberculosis* (*Map*) (10 bacilli/macrophage). Amplification slopes ranged between −3.48 and −3.59 with a correlation coefficient (*R*^*2*^) of 0.992–0.997 and an efficiency of 90.85−93.87%. Data is represented as picograms in bar plots as mean values and standard deviations (*n* = 6).

*Map DNA* was quantified in CaMØs and supernatants from vaccinated and non-vaccinated goats. Control (C-), non-infected CaMØs and its supernatants from vaccinated and non-vaccinated groups proved to be negative. Regarding the infected CaMØs, the quantity of *Map* DNA inside CaMØs from vaccinated goats was an average 5 times higher than in their supernatants. In contrast, CaMØs from non-vaccinated animals showed a quantity of *Map* DNA 5 times lower than in their supernatants (Figure 2). Besides, when comparing between groups, vaccinated goats showed an average of 5 times higher *Map* DNA quantity within CaMØs than non-vaccinated. In contrast, *Map* DNA detected in supernatants was 5 times higher in the non-vaccinated group than in vaccinated (Figure 2). However, statistical differences were not found in any comparisons studied (*P* > 0.05), possibly due to the high individual variability observed between animals from the same group (Figure 2) (Additional file 3).

### Quantification of CaMØs containing *Map* by epifluorescence microscopy

**Figure 3:**
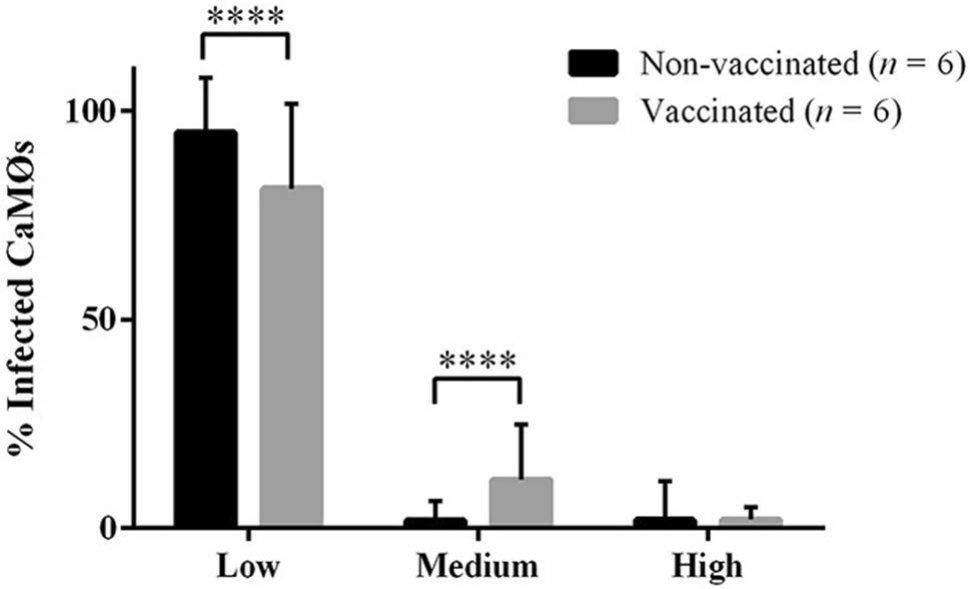
**Percentage of Map-infected CaMØs belonging to the “low”, “medium” and “high” categories.** Proportions were calculated by dividing the number of CaMØs of each category by the total number of infected cells. Data from non-vaccinated and vaccinated goats are represented as means and standard deviations (*n* = 6). *p < 0.05; **p < 0.01; *** *p* < 0.001; ****p < 0.0001.

The mean percentage of infected CaMØs from vaccinated goats was significantly greater (48 ± 24.3%) than from non-vaccinated goats (20.5 ± 16.2%) (*P* < 0.0001). In both groups, most of the infected CaMØs were classified within the “low” category (Figure 3), however, the percentage of CaMØs belonging to this category was significantly higher in non-vaccinated goats (94.75 ± 13.16%) (*P* < 0.0001) than in vaccinated (81.36 ± 20.34%) (Figure 3). On the other hand, the proportion of CaMØs belonging to the “medium” category was significantly higher in the vaccinated group (11.5 ± 13.21%) compared to the non-vaccinated group (1.87 ± 4.73%) (*P* < 0.0001) (Figure 3). No statistical differences between groups were found within the “high” category (2.02 ± 9.33% in non-vaccinated and 2.05 ± 2.97% in vaccinated) (*P* > 0.05) (Figure 3).

### Cytokine expression of infected CaMØs

**Figure 4:**
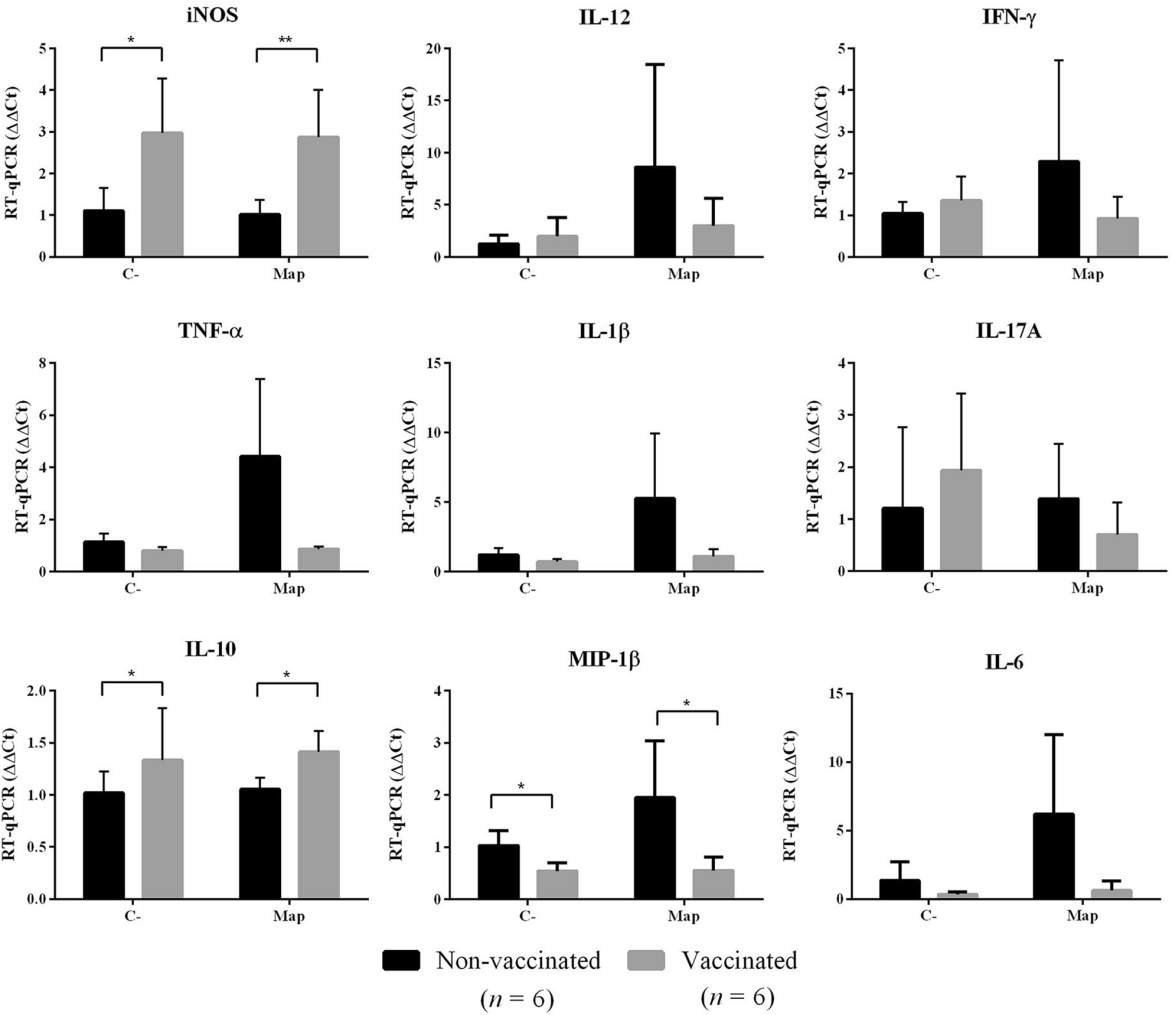
**Cytokine and iNOS expression levels of control and Map-infected CaMØs from non-vaccinated and vaccinated goats.** Data were analysed by the 2^−ΔΔCt^ method taking β-actin as housekeeping gene and mean of no infected macrophages from no vaccinated goats as calibrator. Graphs represent mean values whereas vertical lines represent the standard deviation (*n* = 6). *p < 0.05; **p < 0.01; *** *p* < 0.001; ****p < 0.0001.

The in vitro immune response was characterized by analysing the RNA transcription of IL-10, IL-12, IFN-γ, TNF-α, IL-17A, IL-1β, iNOS, IL-6 and MIP-1β in CaMØs, both infected and non-infected (C-) with *Map*, from non-vaccinated and vaccinated groups (Figure 4). None of RNA samples showed a significant decrease in the β-actin gene expression, suggesting an equivalent RNA loading and the good yield of this gene as internal control.

When comparing the expression of non-infected CaMØs (C-) between vaccinated and non-vaccinated animals (Figure 4), significant differences were found in the transcription levels of iNOS and IL-10 and MIP-1β. The transcription of iNOS and IL-10 was increased in the vaccinated animals (2.96-fold and 1.31-fold increase, respectively) while MIP-1β was decreased in the vaccinated group (1.89-fold decrease).

Similar differences were found when analysing transcription levels in infected CaMØs, as significant higher transcription levels of iNOS (2.83-fold increase) and IL-10 (1.34-fold increase) were found in macrophages from vaccinated goats in comparison with non-vaccinated (*P* < 0.01 and *P* < 0.05, respectively), while MIP-1β transcription was significantly lower (3.52-fold increase) in the non-vaccinated group (*P* < 0.05) (Figure 4).

In addition, but without statistical significance, differences were also observed in the transcription of pro-inflammatory cytokines IL-6, TNF-α, IL-1β, IL-12, IFN-γ and IL-17A, especially when analysing the results from infected CaMØs, where a greater transcription was found in the non-vaccinated group compared with vaccinated one. The high individual variation probably caused the lack of statistical significance in those differences (Figure 4).

## Discussion

Paratuberculosis control programs based on vaccination with heat-killed vaccines such as Silirum^®^ or Gudair^®^ have shown beneficial effects on reducing the severity of clinical signs, lesions and deaths in infected animals [8]. Despite the proved efficacy of this measure, some vaccinated animals still developed severe lesions and/or remained infected and might shed *Map* [10]. These failures in protection are still a main concern on vaccination programs and are a reflection of the lack of knowledge in the mechanisms of the immune response taking part in the protection elicited by vaccination. In this regard, the role of macrophages is especially relevant in the host/pathogen interaction, as they are a key player in the pathogenesis of this disease [43].

Macrophages are the key target cells for *Map* and it has been reported that this mycobacterium is able to survive and grow for several days within MDMs isolated from healthy naïve cattle [14, 44]. Here, CaMØs from both vaccinated and non-vaccinated goats were able to phagocyte *Map*. However, *Map* DNA quantification results revealed that CaMØs from vaccinated goats seem to be more active in phagocytosis, increasing the number of engulfed bacteria according to the *Map*-DNA detected in CaMØs and in their supernatants. However, the qPCR technique does not allow to differentiate the DNA of alive and dead bacteria [45], so the viability count helped to complement *Map* quantification results. Bearing in mind that a significant reduction in *Map* viability was observed in CaMØs from vaccinated goats compared with non-vaccinated ones, it is tempting to hypothesize that a greater microbiocidal activity of CaMØs was induced by the vaccination in these goats. These results contrast with those obtained by Pooley et al. [26] in which ovine monocytes did not show differences in the killing ability between Gudair^®^-vaccinated and non-vaccinated sheep determined by qPCR and viability count. These differences might be explained by (i) the animal species (sheep vs goat), (ii) the use of monocytes instead of macrophages that differ in their phagocytic and microbicidal activity [46], (iii) the co-culture with PBMCs that can modulate the immune response, (iv) the different response window (30 days vs 1 year) and (v) the different vaccine. However, the low number of viable *Map* in all animals is striking (Additional File 3) despite of the initial amount of *Map* infection inoculum. This could be related with a decrease in the viability after being engulfed into the CaMØs (killing effect) and/or after thawing step prior to infection. Nevertheless, since all CaMØs were infected with the same inoculum under the same conditions, differences between groups could not be explained by differences in the inoculum.

Supporting these results, differences in the percentage of infected CaMØs between groups were also observed by immunofluorescence labelling (48% in vaccinated and 20% in non-vaccinated goats) together with the presence of a higher number of *Map-*bacilli detected inside CaMØs from vaccinated animals. When Gollnick et al. [39] compared between animals tested (by fecal culture and ELISA) negative (non-exposed) and positive (previously exposed) to paratuberculosis infection without clinical sings, no differences neither in the percentage of infected MDMs nor in the number of bacteria per cell were observed. Natural infection with *Map* showed differences in the cellular and humoral response between exposed and non-exposed animals [47]. These differences between MDMs from naturally exposed and vaccinated animals could be explained by the fact that vaccination is able to generate a stronger and more effective immune response than the natural sensitization with *Map* on account of the continuous contact with the pathogen and the potentiating action of the adjuvant [7, 48].

Vaccination also influenced the transcriptional levels of pro-inflammatory iNOS and MIP-1β and anti-inflammatory IL-10. CaMØs from vaccinated goats showed a marked up-regulation of iNOS and slighter increase of IL-10 in both C- and infected CaMØs, whereas expression level of MIP-1β was significantly up-regulated in CaMØs from non-vaccinated goats. In contrast, no statistically significant differences were detected between vaccinated and non-vaccinated goats in the transcription levels of IL-12, IFN-γ, TNF-α, IL-1β, IL-17A and IL-6 by CaMØs.

iNOS has been implicated in the modulation of the Th1 and Th2 response and the production of the reactive molecule nitric oxide [49, 50] that has been involved in the killing and growth inhibition of mycobacteria [51]. In fact, the up-regulation of this enzyme in CaMØs may be contributing to the enhancement of phagocytosis and reduction of *Map* viability, and hence, the increase of intracellular killing observed in CaMØs from vaccinated goats [50]. The higher expression of iNOS has been previously described in focal lesions with none or low presence of *Map*, suggesting that it participated in limiting intracellular *Map* growth in relation with classically activated macrophages (M1) [19]. Weiss et al. [44] demonstrated that the infection of MDMs with mycobacteria induces an increase of iNOS expression after 24 h of incubation that was higher in classically activated MDMs. Classical activation of MDMs from naturally resistant cows by supplementation of cell cultures with IFN-γ and/or LPS resulted in a greater expression of iNOS by these MDMs, higher phagocytic index and lower mycobacteria viability suggesting a better microbiocidal activity [52].

Conversely, IL-10 has been related with the survival of *Map* due to its inhibitory effect over the pro-inflammatory cytokines IL-12 and IFN-γ, suppressing the M1 activation of macrophages [53]. Furthermore, studies employing MDMs as in vitro experimental models have shown that *Map* was able to induce the expression and secretion of IL-10 and TGF-α, both of them down-regulating IFN-γ expression levels [32], whereas its neutralization has been related with a higher expression of pro-inflammatory TNF-α, IL-12, IL-8, MHC-II and production of nitric oxide and the intracellular killing of *Map* [54]. IL-10 can be present together with pro-inflammatory cytokines as a regulatory mechanism preventing host tissue damage [55]. In addition, it is known that vaccination with killed whole-cell vaccine induces strong cellular and humoral responses [56]. Thus, it is feasible that protective immunity to *Map* may require the development of a tightly regulated anti and pro-inflammatory response. In fact, a study carried out by Coussens et al. [57] reported that PBMCs from paratuberculosis sub-clinically infected cows had higher transcription levels of IL-10 after in vitro infection with *Map* than clinically infected cows. This suggests that the role of this cytokine in the effective protection against paratuberculosis consists in the control of the pro-inflammatory response, and hence, the local tissue damage. Therefore, it can be hypothesized that increased IL-10 expression by CaMØs from vaccinated animals as reported here, could reflect a key role in controlling the inflammatory response, for instance, by regulating the release of pro-inflammatory cytokines and chemokines that drive the recruitment of other macrophages acting as a niche favouring intracellular multiplication of *Map* and hence the progression and severity of the lesions [13, 57].

Despite the fact that TNF-α, IL-1β, IL-6 and IFN-γ cytokines have been implicated in the in vitro activation of Th1 immune response, the destruction of *Map* and the development of characteristic granulomas [58–61], the current study found no significant influence of vaccination or infection over the transcription of these cytokines by CaMØs, presumably due to the high individual variability. Previous in vitro studies have shown the effect of IFN-γ on the acidification and maturation of phagosomes in MDMs that reduce *Mycobacterium avium* subsp. *avium*, *Mycobacterium bovis* and *Map* viability [52, 62]. In this regard, some studies have reported the potential effect of adherent lymphocytes to tissue plastic plate culture in the macrophages, such as the production of IFN-γ [63] that might modulate the in vitro immune response. Here, the purity of CaMØs, was ~90% [30] and it was verified through the use of CD14, MHC-II and CD11b antibodies. Thus, lack of in vitro supplementation with IFN-γ or the scarce presence of other adherent cells that modulate the host immune response, such as lymphocytes or natural killer cells, [64, 65], give rise to the absence of specific signals associated with their environment that may limit the variations on cytokine transcription from pure CaMØs culture.

In this sense, Silirum^®^ vaccination has been associated with the establishment of a persistent cellular mediated immune response characterized by the increase of IFN-γ production by peripheral blood lymphocytes [8]. Therefore, it can be inferred that paratuberculosis vaccination could trigger the prior activation of peripheral blood monocytes, possibly via IFN-γ stimulation by Th1 lymphocytes present in the blood, and consequently increase their phagocytic ability after in vitro maturation of CaMØs.

Vaccination seemed to exert a stronger influence over cytokine transcription than infection of the CaMØs, as in both groups (vaccinated and non-vaccinated), no significant differences in transcription levels were observed between infected and C- CaMØs in any of the studied cytokines, although IL-12, IFN-γ, TNF-α, IL-1β, MIP-1β and IL-6 tend to be up-regulated in CaMØs infected with *Map*. In addition to the absence of lymphocytes whose presence is related to the modulation of expression and production of theses cytokines, the lack of statistical significance in these differences could be due to the high individual variability between animals of the same group. As mentioned previously, and despite their efficacy in controlling the disease at heard/flock level, commercially available vaccines not only are unable to confer sterile immunity in all cases [6, 7] but vaccination failure could also result in severe, multibacillary lesions, linked to bacteria shedding and death, both in natural [9] and experimentally infected cases [66]. The reasons behind these vaccination failures in some animals are yet unknown but preliminary studies detected differences in the immune response in those individuals where vaccination failed to protect against *Map* infection [66]. Individual differences on how CaMØs react against *Map* have also been observed in the present study, as in the vaccinated group there were great differences in *Map* viability. CaMØs from some animals showed lower number of infected macrophages and these with a lower bacterial load compared to other animals from the same group. It has been described that the macrophages from naturally-resistant individuals showed a higher phagocytic and microbicidal activity against *Mycobacterium bovis* than susceptible ones [52]. Thus, the genetic background of the host could most definitely influence the immune response developed by macrophages against *Map*. For instance, certain polymorphisms in the solute carrier family 11 member A1 gene (SLC11A1), protein that favours the elimination of bacteria, have been associated with susceptibility to *Map* infection [67, 68] since a significant transcription level of SLC11A1 has been detected in MDMs from resistant animals.

Recently, the term trained immunity has been used to refer to a non-specific response to a secondary infection against a related or non-related microorganism mediated by the innate immune system [69, 70]. This process happens independently of T or B lymphocytes and is the result of epigenetic reprogramming of innate immune cells after the first contact with a pathogen, either through infection or vaccination, occurring in bone marrow, peripheral blood and in the tissue through modifications in the intracellular signalling and metabolic response [71]. These changes could lead to the modification of pattern recognition receptors (PRRs) in “trained” macrophages that are involved in the rapid pathogen recognition and an increased protection against secondary pathogens [70].

The most studied stimulus of trained immunity is that caused by Bacillus Calmette-Guerín (BCG) vaccination in humans [72, 73] which has been related with an enhanced function and growth inhibition of circulating monocytes against reinfection and non-related pathogens such as *Staphylococcus aureus* and *Candida albicans* [74, 75]. In the present study, the protective role of paratuberculosis vaccination against non-related pathogens such as *S. aureus* has not been assessed; however, in the light of the results, the role of a trained immunity cannot be ruled out given the increased response of CaMØs from vaccinated animals to reinfection with *Map*. Furthermore, since during *Map* infection there is a recruitment of peripheral blood monocytes at the site of infection that could have been previously “trained”, the possible role of this response is relevant for the understanding of the paratuberculosis outcome after vaccination.

The current study has used CaMØs as an in vitro experimental model for investigating the interaction between *Map* and macrophages in goats vaccinated with Silirum^®^. In this sense, this study has found that vaccination could predispose to a greater capacity of phagocytosis of *Map* and the reduction of its viability in CaMØs. In addition, vaccination also promoted the transcription of IL-10 and iNOS in CaMØs, suggesting that the effective protection conferred by vaccination not necessarily depends on a pro-inflammatory response but also on an anti-inflammatory response and its balance. The high individual variability observed in this study may be related to the variable response observed in in vivo vaccination studies. Further studies aimed at evaluating the role of other variables such as individual genetic variations or different *Map* strains could contribute to explain in more detail the changes in the macrophage function and immune response elicited by vaccination.

## Supplementary Information

**Additional file 1: Schematic illustration of the experimental design. **CaMØs from non-vaccinated and vaccinated goats were culture in 24-well plates and infected with *Map* (MOI 10:1) for 24 h. Control non-infected and Map-infected wells were used by duplicate for each analysis. Image created using Biorender.

**Additional file 2: Sequences of primers used for cytokine RT-qPCR and standard curve data.**

**Additional file 3:Results of the count of viable colony forming units (CFU) and quantification of *Map*-DNA by qPCR performed in CaMØs and supernatants from non-vaccinated and vaccinated animals. **

## Abbreviations

CaMØs: Caprine monocyte-derived macrophages
C-: Non-infected CaMØs
*Ct*: Threshold cycles
*Map*: *Mycobacterium avium* Subsp. *Paratuberculosis*
MDM: Monocyte-derived macrophages
MJ: Mycobactin J
MOI: Multiplicity of infection
OADC: Oleic acid-albumin-dextrose-catalase
PBMCs: Peripheral blood mononuclear cells
RT-qPCR: Reverse transcriptase quantitative polymerase chain reaction
*Tm*: Dissociation peak
