# Supplementary figures and images for: Early response of monocyte-derived macrophages from vaccinated and non-vaccinated goats against in vitro infection with Mycobacterium avium subsp. paratuberculosis
Source: Vet Res. 2021 May 12;52:69. doi: 10.1186/s13567-021-00940-y (PMC8117269; doi:10.1186/s13567-021-00940-y)

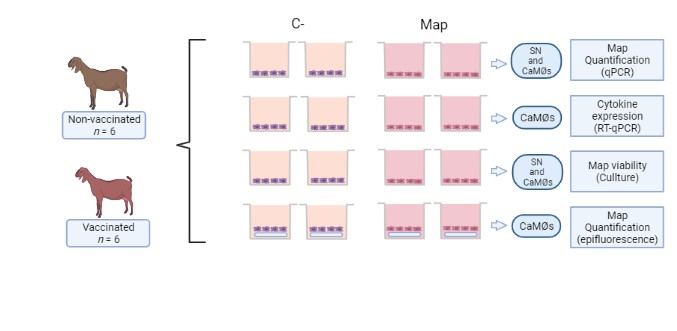

Supplement: Supplementary file 1 — Additional file 1: Schematic illustration of the experimental design. CaMØs from non-vaccinated and vaccinated goats were culture in 24-well plates and infected with Map (MOI 10:1) for 24 h. Control non-infected and Map-infected wells were used by duplicate for each analysis. Image created using Biorender. [file 13567_2021_940_MOESM1_ESM.jpeg]
