# Supplementary material for: Early response of monocyte-derived macrophages from vaccinated and non-vaccinated goats against in vitro infection with Mycobacterium avium subsp. paratuberculosis
Source: Vet Res. 2021 May 12;52:69. doi: 10.1186/s13567-021-00940-y (PMC8117269; doi:10.1186/s13567-021-00940-y)
Supplement: Supplementary file 2 — Additional file 2: Sequences of primers used for cytokine RT-qPCR and standard curve data. [file 13567_2021_940_MOESM2_ESM.docx]

| **Sequences of primers used for cytokine and iNOS RT-qPCR and standard curve data** | | | | | | |
| --- | --- | --- | --- | --- | --- | --- |
| **Target^a^** | **Primer** | **Primer sequences (5’-3’)** | **Product size (bp)** | ***R*^2 b^** | **Slope^c^** | **Reference** |
| IFN-γ  (X52640.1) | QIFN-Fw | GATTCAAATTCCGGTGGATG | 110 | 0.967 | -3.7 | [41] |
|  | QIFN-Rv | TTCTCTTCCGCTTTCTGAGG |  |  |  |  |
| IL-10 (NM_001009327.1) | QIL10-Fw | TGCTGGATGACTTTAAGGGTTACC | 60 | 0.996 | -3.9 | [41] |
|  | QIL10-Rv | AAAACTGGATCATTTCCGACAAG |  |  |  |  |
| TNF-α (NM_001024860.1) | QTNFα-Fw | CCAGAGGGAAGAGCAGTCC | 126 | 0.978 | -3.6 | [41] |
|  | QTNFα-Rv | GGAGCGCTGATGTTGGCTAC |  |  |  |  |
| IL-12 (NM_001009438.1) | QIL12p40-Fw | ATGGAATTTGGTCCACTGATATT | 95 | 0.994 | -3.6 | [41] |
|  | QIL12p40-Rv | GTGAAGTGTCCAGAATAATCCTTT |  |  |  |  |
| IL-1β (NM_001009465.2) | QIL1β-Fw | ACCCCAAAGTCTACCCCAAG | 99 | 0.998 | -3.5 | This study |
|  | QIL1β-Rv | TGAGTCTGTCCTGTACCCTA |  |  |  |  |
| IL17A (XM_004018887.4) | QIL17A-Fw | AGTCTGGTGGCTCTTGTGAA | 113 | 0.996 | -3.4 | This study |
|  | QIL17A-Rv | TTAACGATGTTCAGGTTGAC |  |  |  |  |
| IL-6  (X_68723.1) | QIL6-Fw | CTGGGTTCAATCAGGCGATT | 150 | 0.990 | -3.4 | This study |
|  | QIL6-Rv | GGATCTGGATCAGTGTTCTGA |  |  |  |  |
| iNOS  (XM_013971952.2) | QiNOS-Fw | CTTTTGGCAACGGAGACTC | 125 | 0.992 | -3.5 | This study |
|  | QiNOS-Rv | CTGAGGGTACATGCTGGA |  |  |  |  |
| MIP-1β  (NC_040262.1) | QMIP1β-Fw | CGAATCTCTCCTCTGCCAAG | 193 | 0.991 | -3.6 | This study |
|  | QMIP1β-Rv | ATCCCCGAATGCTACTGTTG |  |  |  |  |
| β-actina (NM_001009784.1) | QBACTIN-Fw | ACACCGCAACCAGTTCGCCAT | 216 | 0.996 | -3.6 | [40] |
|  | QBACTIN-Rv | GTCAGGATGCCTCTCTTGCT |  |  |  |  |
| ^a^ NCBI accession numbers are for ovine cDNA sequences used in primer design. Sequences were checked for similarities with *Capra hircus* sequences using basic local alignment search tool BLAST.  ^b^ Mean minimum coefficient of regression (*R^2^*) of standard curves  ^c^ Mean of standard curve slopes | | | | | | |
