# Supplementary material for: Early response of monocyte-derived macrophages from vaccinated and non-vaccinated goats against in vitro infection with Mycobacterium avium subsp. paratuberculosis
Source: Vet Res. 2021 May 12;52:69. doi: 10.1186/s13567-021-00940-y (PMC8117269; doi:10.1186/s13567-021-00940-y)
Supplement: Supplementary file 3 — Additional file 3:Results of the count of viable colony forming units (CFU) and quantification of Map-DNA by qPCR performed in CaMØs and supernatants from non-vaccinated and vaccinated animals. [file 13567_2021_940_MOESM3_ESM.docx]

| **Results of the count of viable colony forming units (CFU) and quantification of *Map-*DNA by qPCR performed in CaMØs and supernatants from non-vaccinated and vaccinated animals.** | | | | | | |
| --- | --- | --- | --- | --- | --- | --- |
| **Animal** | **Treatment** | **Nº viable CFU** | | **DNA quantity (pg)** | |  |
|  |  | **CaMØs** | **Supernatants** | **CaMØs** | **Supernatants** |  |
| C1 | Non-vaccinated | 880 | 20 | 1.480 | 0.120 |  |
| C2 | Non-vaccinated | 879 | 19 | 0.013 | 0.483 |  |
| C3 | Non-vaccinated | 1373 | 35 | 0.384 | 4.162 |  |
| C4 | Non-vaccinated | 2140 | 70 | 1.348 | 7.973 |  |
| C5 | Non-vaccinated | 833 | 10 | 0.005 | 0.002 |  |
| C6 | Non-vaccinated | 1013 | 35 | 0.150 | 5.924 |  |
| C7 | Vaccinated | 28 | 2 | 0.050 | 0.426 |  |
| C8 | Vaccinated | 80 | 5 | 7.760 | 0.942 |  |
| C9 | Vaccinated | 396 | 5 | 9.330 | 0.733 |  |
| C10 | Vaccinated | 58 | 5 | 4.216 | 1.292 |  |
| C11 | Vaccinated | 10 | 0 | 0.010 | 0.003 |  |
| C12 | Vaccinated | 52 | 0 | 0.030 | 0.011 |  |
